# Recent Advances in Predicting Protein S-Nitrosylation Sites

**DOI:** 10.1155/2021/5542224

**Published:** 2021-02-09

**Authors:** Qian Zhao, Jiaqi Ma, Fang Xie, Yu Wang, Yu Zhang, Hui Li, Yuan Sun, Liqi Wang, Mian Guo, Ke Han

**Affiliations:** ^1^School of Computer and Information Engineering, Heilongjiang Provincial Key Laboratory of Electronic Commerce and Information Processing, Harbin University of Commerce, Harbin 150028, China; ^2^School of Pharmacy, Center of Pharmaceutical Engineering and Technology, Harbin University of Commerce, Harbin 150076, China; ^3^Department of Neurosurgery, The Second Affiliated Hospital of Harbin Medical University, Harbin 150086, China

## Abstract

Protein S-nitrosylation (SNO) is a process of covalent modification of nitric oxide (NO) and its derivatives and cysteine residues. SNO plays an essential role in reversible posttranslational modifications of proteins. The accurate prediction of SNO sites is crucial in revealing a certain biological mechanism of NO regulation and related drug development. Identification of the sites of SNO in proteins is currently a very hot topic. In this review, we briefly summarize recent advances in computationally identifying SNO sites. The challenges and future perspectives for identifying SNO sites are also discussed. We anticipate that this review will provide insights into research on SNO site prediction.

## 1. Introduction

**Figure 1:**
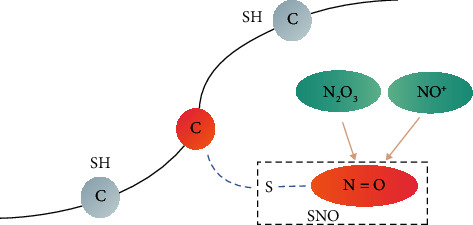
A schematic diagram of protein S-nitrosylation sites. Protein fragments have many residues, of which C (cysteine) is depicted as a circle. When NO and cysteine residues are covalently modified, SNO is formed, which is represented by a warm color, and the rest is gray.

Protein S-nitrosylation (SNO) is one of the most important and common posttranslational modifications (PTMs), as shown in Figure 1, incorporating the covalent modification of nitric oxide (NO) and its derivatives and cysteine residues [1]. Numerous studies have shown that S-nitrosylation regulates multiple physiological and pathological processes, such as the immune response [2], cellular senescence [3], transcription, and posttranslational regulation [4]. In addition, abnormalities in protein S-nitrosylation and other posttranslational modifications can also lead to many diseases, such as Alzheimer's disease [5–7] and breast cancer [8]. In recent years, through molecular recognition and labelling of SNO sites in proteins, many large-scale proteomics experimental screenings have been completed, and the number of SNO proteins verified by experiments is also increasing [9, 10]. As to other protein posttranslational modification sites [11–18], the predicted SNO sites are time-wasting, strenuous, and extortionate through large-scale experimental screening methods. With continuous breakthroughs in sequence and structural biology, computational biology using machine learning has become an indispensable part of drug development [19–36].

As an alternative to biochemical experiments, identifying SNO sites in biological sequences with the least cost and efficiency in recent years is a focus of current research. To help researchers understand the development of this field, this review will use Chou's five-step rule as the literature selection criteria [37]: (1) how to select or construct an effective fiducial marker dataset subcellular location to train and test predictors, (2) how to express the sample with an effective formula that can truly reflect the intrinsic correlation between the sample and predicted target, (3) how to introduce or develop powerful algorithms to make predictions, (4) how to correctly conduct cross-validation tests to objectively evaluate the expected prediction accuracy, and (5) how to build a user-friendly web server for forecasters. In addition, to help researchers overcome the overall development of this field, this review briefly introduces early research on the identification of SNO sites using biochemical methods.

## 2. Materials

High-quality datasets are the cornerstone of scientific research [38, 39]. With the development of proteomics and the advancement of research by scientists, the number of experimentally identified SNO sites is also increasing. In the process of predicting S-nitrosylation sites, the dynamic changes of the database and the dataset are sorted in this part.

### 2.1. Database

UniProt [40] (Universal Protein Resource) is a high-quality, extensive, and open-access database of protein sequences and functional annotations created and maintained by the UniProt Consortium, namely, EBI, SIB (Swiss Institute of Bioinformatics), and PIR (Protein Information Resource), an association of three institutions. It mainly includes three parts: the UniProtKB knowledge base, UniParc archive library, and UniRef reference sequence set. The UniProt database collects cysteine SNO sites from different species. With the continuous addition of a large number of experimentally verified SNO sites, the dataset used by scientists to predict SNO sites is also updated accordingly [41, 42].

dbSNO [43] (database of cysteine S-nitrosylation) is the first database specifically designed to integrate experimentally determined SNO sites and their structure or function information. SNO peptide sequences collected from different sources are heterogeneous, so dbSNO maps the identity of these sequences to UniProtKB protein entry. In addition, the dbSNO database also provides powerful structural and functional analysis functions to help researchers better understand the structural correlation and shared motifs of these SNO peptide sequences. The dbSNO database is divided into two versions: the first version ended in April 2012, and this version contains 43,000 experimentally verified SNO peptide sequences collected in numerous published studies using text mining methods; the second version is dbSNO2.0. In this version, dbSNO2.0 is also expanded to explore the structural environment of low SNO sites and the regulatory network resources of S-nitrosylation proteins. In SNO site prediction experiments, many scientists have also used the S-nitrosylation peptide sequence of the dbSNO database [41, 44].

PRISMOID [45] is a newly established database focusing on posttranslational modification and mutations with functional impact. Compared with traditional databases that focus on protein sequences, PRISMOID has added the real 3D structure of proteins and is equipped with various friendly operation interactions for information visualization. This database is the first version and contains 37 kinds of PTM annotation data (323 nitrosylation sites) manually compiled and is expected to be updated at least every 6 months. In addition, PRISMOID also integrates information such as the protein secondary structure and protein disordered regions to facilitate researchers to carry out scientific research.

### 2.2. Datasets

With the continuous in-depth understanding of the characteristics of S-nitrosylation, an increasing number of SNO peptide sequences have been identified, and the datasets used to predict SNO sites are based on previous studies. Dynamic changes are taking place. Therefore, the datasets commonly used by researchers for the detection of S-nitrosylation sites are chronologically explained in this section.

SNOSID, the first bioinformatics tool for predicting S-nitrosylation sites, was developed by Hao et al. [46]. In this study, they used S-nitrosoglutathione-treated rat cerebellar lysates. In 56 of the proteins, 68 cysteine sites were designated, and the initial limited 65 positive and negative samples were selected in the random sampling process. Xue et al. [47] also developed a predictor GPS-SNO for predicting SNO sites. They collected 363 experimentally verified S-nitrosylation sites published on PubMed using nitrosylated or nitrosylation as keywords and then integrated the public database SysPTM [48] and two large-scale S-nitrosylation site surveys [46, 49]. Finally, 504 positive sites and 2581 negative sites were obtained through sequence identity threshold setting [50] and protein sequence alignment [51]. A year later, Li et al. [52] used GPS-SNO datasets to develop a method for predicting SNO sites using SVM. Before long, Lee et al. [53] also developed a tool for predicting SNO sites, SNOSite. In this study, the training set and test set are from Chen et al. [54] and GPS-SNO data, respectively. Chen et al. used a high-throughput S-alkylating biotin conversion method in SNAP/L cysteine-stimulated mouse endothelial cells to obtain 586 positive sites and 2728 negative sites. In addition, since the data came from different datasets, the test set and training set may have the same homology. Therefore, they first defined SNO sequences with more than 30% identity as homologous sequences and then used BLAST 2 [55] to compare the fragment sequences. A test set containing 479 positive sites and 2501 negative sites was finally obtained.

In 2012, Li et al. [56] developed a method to predict and analyse SNO sites using minimal-redundancy-maximal-relevance and incremental feature selection. The dataset used in this experiment had three sources. The first source of SNO sites was the UniProt database [57] (version 2011_07) and the second from GPS-SNO and the third from large-scale S-nitrosylation site surveys [58–61] at that time to obtain the remaining two datasets. Finally, a training set (784 positive sites and 1568 negative sites) and a test set (43 positive sites and 121 negative sites) were obtained. In 2013, Xu et al. [41] developed iSNO-PseAAC, a tool for predicting S-nitrosylation sites. They randomly selected 438 proteins from the dbSNO database, and the sequence identity of these proteins was less than 40%. After comparison with the annotations in the dbSNO database, 731 positive sites and 810 negative sites verified by experiments were collected in the UniProt database [62] (version 2012_08). Xu et al. [63] improved on the basis of iSNO-PseAAC and developed iSNO-AAPair. This experiment used the original data of S-nitrosylation sites from dbSNO (version 1.0) and the UniProt database (version 2012_08). By using Chou's peptide formula [64–67], sequence identity setting, and random selection, 2300 SNO-positive and SNO-negative sites verified by experiments were obtained as training sets, and 81 positive and 100 negative sites were obtained as test sets.

In 2014, Jia et al. [68] developed a bioinformatics tool named iSNO-ANBPB used to predict SNO sites. In this experiment, they used the dataset constructed by Li et al. [52] and iSNO-PseAAC and obtained 1229 positive sites and 1223 negative sites by sequence identity setting and clustering. Soon, Zhang et al. [44] also developed the experimental tool PSNO. To reach a consensus assessment with previous experiments, they first constructed a training set containing 731 positive and 810 negative loci and a test set containing 53 positive and 103 negative sites from the dbSNO database. In addition, the 2302 positive sites selected from the GPS-SNO dataset and the 81 positive sites and 100 negative sites selected from the iSNO-PseAAC dataset were used as the test set.

After a brief stagnation, Xie et al. [69] used deep learning technology to develop a bioinformatics tool, DeepNitro, to predict SNO sites. They searched the relevant literature published before June 30, 2015, from PubMed and obtained a training set containing 20862 sites (3409 positive sites and 17453 negative sites) through residue modification and sequence clustering. To reach a consensus with previous research, they collected the latest data and eliminated the repeated sequences in previous work. Finally, an independent test set was built (485 positive sites and 4947 negative sites).

In 2019, Li et al. [70] predicted S-nitrosylation sites by multifeature fusion. In this study, they used 731 positive sites and 810 negative sites of iSNO-PseAAC and iSNO-AAPair as the training set and 43 positive sites and 121 negative sites of Li et al. as the test set. At the same time, Hasan et al. [71] developed PreSNO and used the DeepNitro dataset. To avoid overestimation of the prediction model, CD-HIT was used to screen homology and eliminate SNO sequences with the same window. Furthermore, to avoid prediction bias, they adopted the method of randomly taking and merging the sequences to balance the number of SNO-positive and SNO-negative sites.

## 3. Research Review

For protein S-nitrosylation site prediction, the traditional method is based on biochemical methods, but the SNO sites predicted are time-wasting, strenuous, and extortionate. With continuous breakthroughs in sequence and structural biology, computing methods have gradually become the mainstream of current research. This method is low cost and efficient. This section focuses on computational methods based on machine learning or deep learning to provide researchers with a systematic understanding of the development of this field. Traditional biochemical methods are also briefly introduced.

### 3.1. Biochemical Methods

Jaffrey and Snyder [72] invented biotin switch assay (BSA) technology. This method first converts nitrosylated cysteine residues into biotinylated cysteine residues and then detects biotin or specific proteins by Western blot [73] to detect the proteins labelled by biotin. BSA not only greatly improves the feasibility of SNO protein identification but also promotes the improvement of high-throughput identification of SNO sites. In 2005, Gao et al. [74] proposed using BSA and protein sequencing technology to identify endogenous SNO sites. The method is simple and rapid and can meet the needs of separation, purification, and identification of SNO proteins.

In 2006, Hao et al. [46] extended the original biotin method and proposed a new improved method, SNOSID. SNOSID introduced a protein hydrolysis and digestion step before capturing the antibiotin protein. This step was not like the previous complete separation of the peptide fragment of SNO protein but the selective separation of the residues containing the SNO site before. SNOSID also introduced the machine learning algorithm SVM for the first time. In addition, the original limited 65 positive samples and 65 negative samples as training data, but the prediction results were not ideal.

Although SNOSID technology can identify the target proteins and target sites of S-nitrosylation, the degree of protein nitrosamine cannot be accurately measured. With the advancement of proteomics technology, Wu et al. [75] and Fares (2014) developed a technology combining BSA with an isotope-coded affinity tag (ICAT). This technique was the first to achieve large-scale identification of S-nitrosylation residues but is disadvantaged by its use of isotopes.

### 3.2. Computational Biology Methods

With the continuous emergence of massive biological sequences in the postgene era, traditional biochemical sequencing methods are far from being able to meet the needs of development. However, machine learning algorithms cannot directly deal with biological sequence data. Therefore, how to use discrete models or a certain way to express biological sequences and fully express their sequence information or key pattern features has become the focus and content of research in computational biology [76–84]. Since Chou proposed the pseudoamino acid composition [85, 86] or PseAAC [87], computational biology based on machine learning or deep learning has also developed rapidly. The following introduces the software and server based on Chou's five-step rule to predict protein S-nitrosylation sites through algorithms. See Table 1 for details.

#### 3.2.1. GPS-SNO

Xue et al. [47] developed GPS-SNO1.0, a tool for predicting protein S-nitrosylation sites using the GPS3.0 algorithm. The software is developed on the basis of the GPS2.0 algorithm [88] previously proposed. In this study, they first used the amino acid substitution matrix to calculate the nitrosylation peptide sequence and obtain the corresponding score. Then, *k*-means clustering, peptide selection (PS), weight training (WT), and matrix mutation (MaM) were used to improve the performance. The accuracy of the experiment under low threshold conditions was 75.80%, the sensitivity was 53.57%, and the specificity was 80.14%. In addition, the prediction ability of GPS-SNO on 485 potential S-nitrosylation low positions was also tested, and 371 positions of these targets were successfully predicted. GPS-SNO can be obtained for free from the website http://sno.biocuckoo.org/.

#### 3.2.2. CPR-SNO

GPS-SNO has initially explored its ability on S-nitrosylated substrates. Although good results have been achieved, there is still room for improvement. Li et al. [52] developed CPR-SNO. In this study, they used SVM as a classifier and used the coding scheme based on coupling mode to realize the prediction system. In the performance evaluation, the *F*-score is used to identify the effective coding scheme, and referencing the work of Xue et al. [47], tenfold cross-validation is used for verification. In addition, this research solves the problem of existing coding schemes not being able to provide enough information to predict SNO sites. By using the *F*-score to identify effective coupling modes, they proved that some coupling modes are not related to S-nitrosylation. The CPR-SNO server is no longer in use.

#### 3.2.3. SNOSite

Although traditional research on the characteristics and mechanism of S-nitrosylation has made great progress, the understanding of its substrate specificity is still insufficient. In 2011, Lee et al. [53] made a breakthrough on this issue and developed a new bioinformatics tool, SNOSite, for predicting SNO sites. In this study, they used maximal dependence decomposition (MDD) to serialize the nitrosylation sites into different subgroups and used SVM to generate a prediction model for each MDD cluster motif. By using fivefold cross-validation, the SVM using MDD clustering achieves 90% accuracy. SNOSite can be used for free on the website http://csb.cse.yzu.edu.tw/SNOSite/.

#### 3.2.4. mRMR and IFS Method

Feature selection is useful for machine learning-based biosequence analysis [22, 89–105], including SNO prediction. Li et al. [56] developed a predictor based on the nearest neighbour algorithm [106] (NNA), which uses maximum relevance minimum redundancy [107] (mRMR) for incremental feature selection [108–110] (IFS). In this work, they generated 666 features from the peptide sequences used in the experiment and then used mRMR to rank the relevance and redundancy of these features in order of importance. For the obtained feature rankings, the best features are determined through IFS, and then, these features are constructed into different feature sets. Finally, the predictive evaluation performance of each feature set is generated by NNA. The best feature combination composed of 67 features is selected through the above method, and an accuracy of 0.61607 is obtained in the test set. In addition, this experiment also shows that the characteristics of the site far from the central cysteine can help determine the S-nitrosylation site. There is no online server for this predictor.

#### 3.2.5. iSNO-PseAAC and iSNO-AAPair

Xu et al. [41] proposed a new SNO site predictor iSNO-PseAAC. In this study, they used PseAAC to represent protein sequence information, constructed as a 21 × 20 position-specific amino acid propensity (PSAAP) matrix, and finally used the conditional random field (CRF) algorithm to construct a predictor for predicting SNO sites. The cross-validation test of iSNO-PseAAC on an independent dataset also achieved a success rate of over 90%. iSNO-PseAAC can be obtained for free on the website http://app.aporc.org/iSNO-PseAAC/. However, iSNO-PseAAC simply considers the positional orientation of each group of amino acids when predicting variables but does not consider any correlation between them. The amino acids in all proteins are processed individually. However, there must be some connection between them in physiology or mechanism. To solve this problem, Xu et al. [63] made improvements on the basis of iSNO-PseAAC, added related influences when predicting protein SNO sites, and released a new SNO site prediction tool iSNO-AAPair. It considers the coupling effects of all pairs formed by the closest residues along the protein chain and the pairs formed by the closest residues. The predictor was cross-validated on the latest benchmark test set and achieved good performance. iSNO-AAPair can be obtained for free on the website http://app.aporc.org/iSNO-AAPair/.

#### 3.2.6. iSNO-ANBPB

Jia et al. [68] proposed an iSNO-ANBPB predictor based on support vector machines. In this study, they constructed four feature extraction schemes and combined Chou's pseudoamino acid composition for model evaluation. The cross-validation of the basic SVM showed that the combination scheme using ANBPB for feature extraction obtained the best test results. In addition, studies [56] have shown that examples of the static charge of amino acids in cysteine residues and the secondary structure of amino acids play a key role in the prediction of SNO sites. Therefore, in addition to feature extraction, this study also considered the physical and chemical information in the peptide sequence. There is no online server for this predictor.

#### 3.2.7. PSNO

In 2014, Zhang et al. [44] proposed a new bioinformatics tool, PSNO, for predicting SNO sites. In this study, they studied various derived features of the experimental sequence and integrated them into PseAAC to represent the experimental sample. In addition, to prevent the increase in the amount of information from increasing the difficulty of feature dimensions and predictors [111], they used relative entropy to discard noisy features from the high-level space and then optimize the optimal feature subset. However, the features of the optimal subset are different, so IFS is used here to rank these features, and a classifier based on 10-fold cross-validation is constructed for each of the optimal feature subsets. Finally, the *k*-nearest neighbour algorithm is used to predict the input sample and discriminate the prediction samples. In 10-fold cross-validation, the accuracy of PSNO was 75.67%, and the accuracy of MCC was 0.5119. With the completion of the whole-genome sequencing project, the gap in the sequence structure is rapidly expanding. In the absence of a protein structure, sequence-based prediction represented by PSNO can become a powerful supplement to replace structure-based prediction. The server provided by the software is now invalid.

#### 3.2.8. DeepNitro

Since Hinton et al. [112] proposed the hierarchical training strategy to solve the gradient diffusion problem in 2006, deep learning technology has also been widely used in computational biology [113–126] and drug discovery [127–134]. In 2018, Xie et al. [69] used the deep learning algorithm for the first time to develop the S-nitrosylation site prediction bioinformatics tool DeepNitro. DeepNitro is an eight-layer neural network. The first layer is the data input layer, which is used to assign prediction and training values to neurons; the second to seventh layers are fully connected layers, of which the second to fourth layers use the dropout algorithm to improve the generalization ability of unknown data.

In the process of neural network design, to solve the problem of gradient diffusion in the training process, the ReLU function was used as the activation function, and the log-likelihood probability was used as the loss function to optimize the weights and other parameters in the neural network. In the process of backpropagation, a minibatch gradient descent algorithm is used to update the network parameters. Compared with traditional optimization algorithms, the momentum method is superior in optimizing parameters such as weights, so the momentum method was selected as the optimization function. In addition, L1 and L2 regular terms are introduced as hyperparameters to prevent overfitting. For the last layer, the softmax algorithm is used to obtain the probability distribution of the prediction results. Finally, through principal component analysis (PCA), DeepNitro obtained an AUC value of 0.7437 on the test set. DeepNitro uses deep learning algorithms, new encoding algorithms, and a position-specific scoring matrix [135] (PSSM) to greatly improve the accuracy of nitrosation site prediction and provides a free website server (http://deepnitro. http://renlab.org/) for academic research.

#### 3.2.9. PreSNO

In 2019, Hasan et al. [71] proposed a prediction tool, PreSNO, for predicting protein SNO sites by an ensemble algorithm. The focus of the study was the use of four different coding schemes, including the composition of profile-based amino acids (CPA), *K*-space spectral amino acid composition (SAC), tripeptide composition from the PSSM (TCP), and physical-chemical properties of amino acids (PPA). The four coding schemes use SVM and random forest to calculate the probability score and then multiply it by weight to calculate the prediction effect of PreSNO. Through 5-fold cross-validation, PreSNO also achieved excellent performance. The predictor can be obtained for free on the website (http://kurata14.bio.kyutech.ac.jp/PreSNO/).

#### 3.2.10. Multiple Features Combination Method

Soon, Li et al. [70] proposed a method to predict protein S-nitrosylation sites using multifeature mixing. This work improves prediction performance by extracting nine sequence features, such as parallel correlation pseudoamino acid composition (PC-PseAAC), general parallel correlation pseudoamino acid composition [136], and ANBPB. Then, the importance of amino acids is evaluated by subtracting the given amino acids from the information gain [137] (IG), and finally, the max-relevance-max-distance [138] (MRMD) generates a feature subset with lower redundancy and strong correlation with the target category. In the cross-validation of the test set, the ACC and MCC of this method were 73.17% and 0.3788, respectively, which becomes a useful supplement to the existing SNO identification tools.

## 4. Concluding Remarks and Perspectives

Many physiological and pathological studies of SNO have been reported in recent years. Therefore, accurate prediction of SNO sites will pave the way to speed up related drug development.

Several exciting computational methods have been proposed to predict SNO. Although these works promoted research on SNO and facilitated the prediction of SNO sites, the following challenges should be considered in future works.

Although many predictors have been developed to predict SNO sites, some corresponding indicators have greatly improved the space. This is because existing methods were trained on the basis of an imbalanced dataset. To solve this problem, it is necessary to collect many more positive SNO sites to enlarge the number of SNO sites in the dataset and balance it. In addition, the focus of future research in this field is to use these new technologies and methods to predict more nitrosylated target proteins and sites to reveal the mechanism by which nitrosylation regulates various physiological processes.

## Figures and Tables

**Table 1:** List of 13 predictors for predicting the SNO sites in protein sequences.

No.	Name	Link	Time	Refs
1	SNOSID	Not provided	2006	[46]
2	GPS-SNO	http://sno.biocuckoo.org/	2010	[47]
3	CPR-SNO	http://math.cau.edu.cn/CPR-SNO	2011	[52]
4	SNOSite	http://csb.cse.yzu.edu.tw/SNOSite	2011	[53]
5	Li et al.	Not provided	2012	[56]
6	iSNO-PseAAC	http://app.aporc.org/iSNO-PseAAC	2013	[41]
7	iSNO-AAPair	http://app.aporc.org/iSNO-AAPair	2013	[63]
8	iSNO-ANBPB	Not provided	2014	[68]
9	PSNO	http://59.73.198.144:8088/PSNO	2014	[44]
10	DeepNitro	http://deepnitro.renlab.org	2018	[69]
11	PreSNO	http://kurata14.bio.kyutech.ac.jp/PreSNO	2019	[71]
12	Li et al.	Not provided	2019	[70]
